# RbZnFe(PO_4_)_2_: synthesis and crystal structure

**DOI:** 10.1107/S205698901601046X

**Published:** 2016-07-07

**Authors:** Abdessalem Badri, Mongi Ben Amara

**Affiliations:** a Unité de recherche, Matériaux Inorganiques, Faculté des Sciences, Université de Monastir, 5019 Monastir, Tunisia

**Keywords:** crystal structure, iron phosphate, rubidium, open-framework structure

## Abstract

A new iron phosphate, rubidium zinc iron(III) phosphate, RbZnFe(PO_4_)_2_, has been synthesized as single crystals by the flux method. This compound is isostructural to the previously reported KCoAl(PO_4_)_2_ [Chen *et al.* (1997 ▸). *Acta Cryst.* C**53**,1754–1756]. Its structure consists of a three-dimensional framework built up from corner-sharing PO_4_ and (Zn,Fe)O_4_ tetra­hedra. This mode of linkage forms channels parallel to the [100], [010] and [001] directions in which the Rb^+^ ions are located.

## Abstract 2

Rubidium zinc iron(III) phosphate is isostructural to KCoAl(PO_4_)_2_. Its structure consists of a three-dimensional framework built up from corner-sharing PO_4_ and (Zn,Fe)O_4_ tetra­hedra. This mode of linkage forms tunnels parallel to the [100], [010] and [001] directions in which the Rb^+^ ions are located.

## Chemical context   

Phosphates with open-framework structures, similar to other porous materials such as zeolites, are inter­esting because of their wide industrial and environmental applications ranging from catalysis to ion-exchange and separation (Gier & Stucky, 1991 ▸; Maspoch *et al.*, 2007 ▸). Among them, iron phosphates (Redrup & Weller, 2009 ▸; Lajmi *et al.*, 2009 ▸) are particularly attractive because of their rich crystal chemistry (Moore, 1970 ▸; Gleitzer, 1991 ▸) and they present inter­esting and variable physical properties (Elbouaanani *et al.*, 2002 ▸; Riou-Cavellec *et al.*, 1999 ▸). Among the variety of iron orthophosphates synthesized and characterized over the past three decades, only two rubidium-containing compounds have been reported, namely Rb_9_Fe_7_(PO_4_)_10_ (Hidouri *et al.*, 2010 ▸) and RbCuFe(PO_4_)_2_ (Badri *et al.*, 2013 ▸). In this paper, we report the structure of a new rubidium iron orthophosphate, RbZnFe(PO_4_)_2_, synthesized during our investigation of the Rb_3_PO_4_–Zn_3_(PO_4_)_2_–FePO_4_ quasi-system. This compound is isostructural to KCoAl(PO_4_)_2_ (Chen *et al.*, 1997 ▸) and KZnFe(PO_4_)_2_ (Badri *et al.*, 2014 ▸).

## Structural commentary   

**Figure 1:**
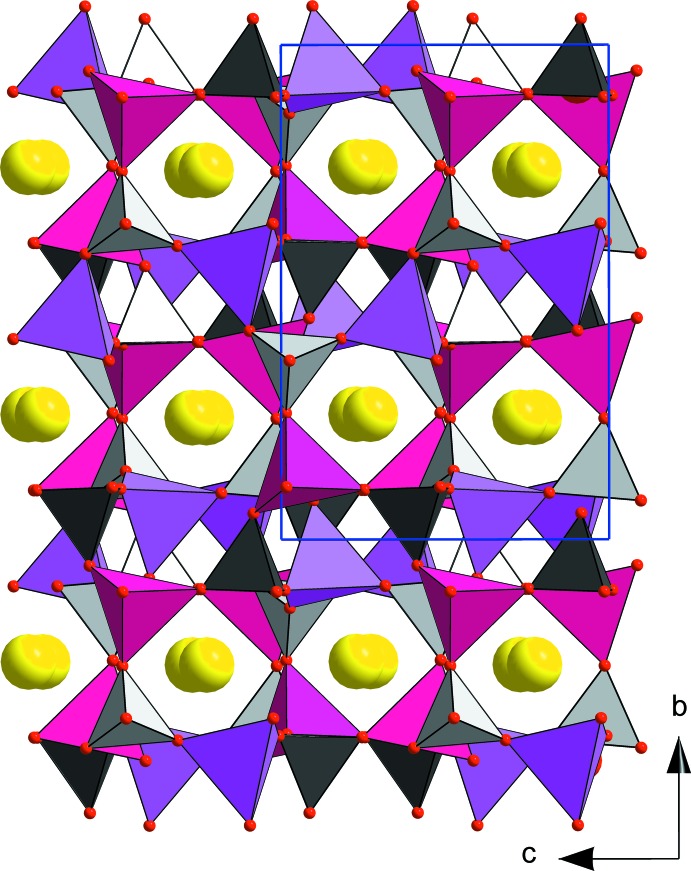
A view of the crystal structure of RbZnFe(PO_4_)_2_ along [100]. Colour key: *M*(1)O_4_ tetrahedra are purple, *M*(2)O_4_ tetrahedra are red, P(1)O_4_ tetrahedra are dark grey, P(2)O_4_ tetrahedra are light grey and Rb^+^ cations are yellow spheres.

**Figure 2:**
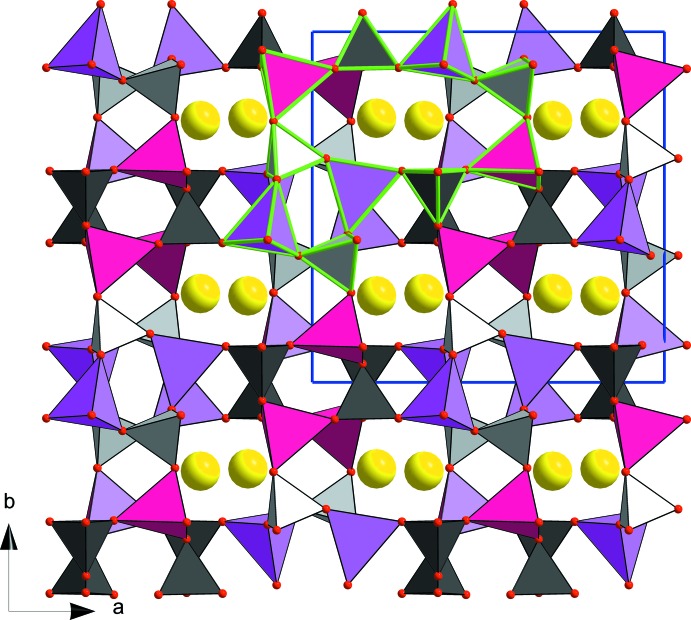
A view of the crystal structure of RbZnFe(PO_4_)_2_ along [001], showing the elliptical and quasi-rectangular forms of corner-sharing *M*O_4_ and PO_4_ tetrahedra (edge with green colour). The colour key is as in Fig. 1.

The structure is made up of a three-dimensional assemblage of *M*O_4_ (*M* = 0.5Zn + 0.5Fe) and PO_4_ tetra­hedra through corner-sharing. This framework delimits crossing channels along the [100] and [001] directions, in which the Rb^+^ ions are located (Figs. 1 ▸ and 2 ▸). A projection of the structure along [001] direction reveals that each *M*O_4_ tetra­hedron is linked to four PO_4_ tetra­hedra by sharing corners. In addition, it shows the presence of two kinds of rings through corner-sharing of *M*O_4_ and PO_4_ tetra­hedra (Fig. 2 ▸). The first presents an elliptical form and comprises four *M*O_4_ and four PO_4_ tetra­hedra, the second consists of two *M*O_4_ and two PO_4_ tetra­hedra and has a quasi-rectangular form. From an examination of the inter-atomic distances (cation–oxygen), the *M*(1) and *M*(2) sites exhibit similar regular tetra­hedral environments, as seen in the cation–oxygen distances which vary from 1.877 (5) to 1.900 (5) Å for *M*(1) and from 1.860 (6) to 1.919 (5) Å for *M*(2). The average distances of 1.885 (2) and 1.888 (2) Å are between the values of 1.926 (2) Å observed for tetra­hedrally coordinated Zn^2+^ ions in the zinc phosphate RbZnPO_4_ (Elammari & Elouadi, 1991 ▸) and 1.865 Å reported for the Fe^3+^ ions with the same coordination in the iron phosphate in FePO_4_ (Long *et al.*, 1983 ▸). The P—O distances within the PO_4_ tetra­hedra are between 1.514 (5) and 1.535 (5) Å and with mean distances of 1.523 (9) Å for P(1) and 1.520 (3) Å for P(2), consistent with the value of 1.537 Å calculated by Baur (1974 ▸) for orthophosphate groups.

**Figure 3:**
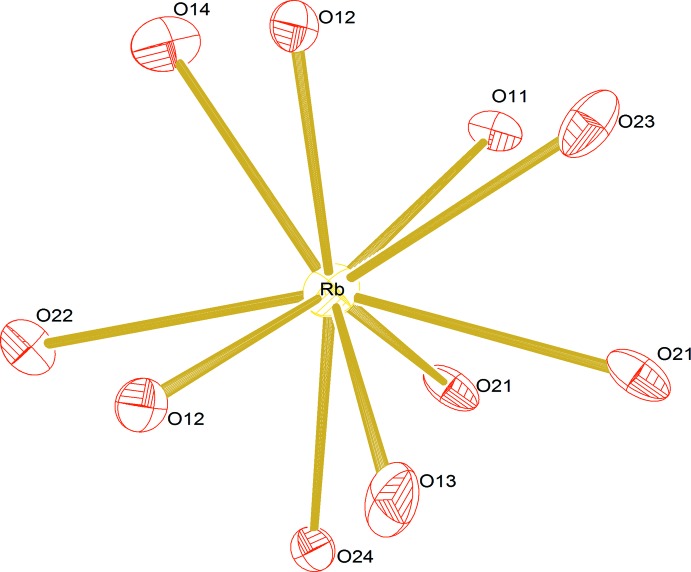
The environment of the Rb cations, showing displacement ellipsoids drawn at the 50% probability level. Authors: Define symmetry operators (in the Figure) and codes (in the caption)

The Rb^+^ ions occupy a single site at the inter­section of the crossing tunnels. Their environment was determined assuming all cation–oxygen distances to be shorter than the shortest distance between Rb^+^ and its nearest cation. This environment (Fig. 3 ▸) then consists of ten O atoms with Rb—O distances ranging from 2.925 (6) to 3.298 (7) Å.

## Synthesis and crystallization   

Single crystals of RbZnFe(PO_4_)_2_ were grown in a flux of rubidium dimolybdate Rb_2_Mo_2_O_7_, in an atomic ratio P:Mo = 4:1. Appropriate amounts of Rb_2_CO_3_, Zn(NO_3_)_2_·6H_2_O, Fe(NO_3_)_3_·9H_2_O, (NH_4_)_2_HPO_4_ and MoO_3_ were used. All of the chemicals were analytically pure from commercial sources and used without further purification. The reagents were weighted in the atomic ratio P:Mo = 2:1 and dissolved in nitric acid and then dried for 24 h at 353 K. The dry residue was gradually heated to 873 K in a platinum crucible to remove the decomposition products. In a second step, the mixture was ground, melted for 1 h at 1173 K and subsequently cooled at a rate of 10 K h^−1^ to 773 K, after which the furnace was turned off. The crystals obtained by washing the final product with warm water in order to dissolve the flux are essentially comprised of beige hexa­gonally shaped crystals of RbZnFe(PO_4_)_2_.

## Refinement   

Crystal data, data collection and structure refinement details are summarized in Table 1 ▸. The application of direct methods revealed the Rb atoms and located two sites, labelled *M*(1) and *M*(2), statistically occupied by the Fe^3+^ and Zn^2+^ ions. This distribution was supported by the *M*(1)—O and *M*(2)—O distances which are between the classical pure Zn—O and Fe—O values. Succeeding difference Fourier syntheses led to the positions of all the remaining atoms.

Despite several synthesis attempts, all the obtained crystals of RbZnFe(PO_4_)_2_ were of poor quality, resulting in the large discrepancies found in a number of reflections; hence in this study the refinement was performed using a filter of the reflections by [sin (θ)/λ]. The four reflections (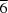
85, 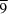
34, 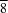
85 and 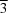
75) were omitted as the difference between the observed and calculated structure factors was larger than 10σ.

## Supplementary Material

Crystal structure: contains datablock(s) global, I. DOI: 10.1107/S205698901601046X/br2261sup1.cif

Structure factors: contains datablock(s) I. DOI: 10.1107/S205698901601046X/br2261Isup2.hkl

CCDC reference: 1488120

Additional supporting information: crystallographic information; 3D view; checkCIF report

## Figures and Tables

**Table 1:** Experimental details

Crystal data	
Chemical formula	RbZnFe(PO_4_)_2_
*M*_r_	396.63
Crystal system, space group	Monoclinic, *C*2/*c*
Temperature (K)	293
*a*, *b*, *c* (Å)	13.601 (4), 13.304 (5), 8.978 (9)
β (°)	100.76 (5)
*V* (Å^3^)	1596.0 (18)
*Z*	8
Radiation type	Mo *K*α
μ (mm^−1^)	11.29
Crystal size (mm)	0.43 × 0.25 × 0.18

Data collection	
Diffractometer	Enraf–Nonius TurboCAD-4
Absorption correction	Part of the refinement model (Δ*F*) (Walker & Stuart 1983 ▸)
*T*_min_, *T*_max_	0.054, 0.070
No. of measured, independent and observed [*I* > 2σ(*I*)] reflections	1409, 1409, 1227
*R*_int_	0.089
(sin θ/λ)_max_ (Å^−1^)	0.594

Refinement	
*R*[*F* ^2^ > 2σ(*F* ^2^)], *wR*(*F* ^2^), *S*	0.036, 0.110, 1.05
No. of reflections	1409
No. of parameters	118
	*w* = 1/[σ^2^(*F*_o_^2^) + (0.0565*P*)^2^ + 31.2735*P*] where *P* = (*F*_o_^2^ + 2*F*_c_^2^)/3
Δρ_max_, Δρ_min_ (e Å^−3^)	0.85, −0.76

Computer programs: *CAD-4 EXPRESS* (Enraf–Nonius, 1994 ▸), *XCAD4* (Harms & Wocadlo, 1995 ▸), *SIR92* (Altomare *et al.*, 1993 ▸), *SHELXL2014* (Sheldrick, 2015 ▸), *DIAMOND* (Brandenburg, 1999 ▸) and *WinGX* (Farrugia, 2012 ▸).

## supplementary crystallographic information

### Crystal data

**Table:**

RbZnFe(PO_4_)_2_	*F*(000) = 1496
*M**_r_* = 396.63	*D*_x_ = 3.301 Mg m^−^^3^
Monoclinic, *C*2/*c*	Mo *K*α radiation, λ = 0.71073 Å
*a* = 13.601 (4) Å	Cell parameters from 25 reflections
*b* = 13.304 (5) Å	θ = 8.1–11.1°
*c* = 8.978 (9) Å	µ = 11.29 mm^−^^1^
β = 100.76 (5)°	*T* = 293 K
*V* = 1596.0 (18) Å^3^	Prism, brown
*Z* = 8	0.43 × 0.25 × 0.18 mm

### Data collection

**Table:**

Enraf–Nonius TurboCAD-4 diffractometer	*R*_int_ = 0.089
Radiation source: fine-focus sealed tube	θ_max_ = 25.0°, θ_min_ = 2.2°
non–profiled ω/2τ scans	*h* = −16→15
Absorption correction: part of the refinement model (Δ*F*) (Walker & Stuart 1983)	*k* = 0→15
*T*_min_ = 0.054, *T*_max_ = 0.070	*l* = 0→10
1409 measured reflections	2 standard reflections every 120 min
1409 independent reflections	intensity decay: 1%
1227 reflections with *I* > 2σ(*I*)	

### Refinement

**Table:**

Refinement on *F*^2^	118 parameters
Least-squares matrix: full	0 restraints
*R*[*F*^2^ > 2σ(*F*^2^)] = 0.036	*w* = 1/[σ^2^(*F*_o_^2^) + (0.0565*P*)^2^ + 31.2735*P*] where *P* = (*F*_o_^2^ + 2*F*_c_^2^)/3
*wR*(*F*^2^) = 0.110	(Δ/σ)_max_ < 0.001
*S* = 1.05	Δρ_max_ = 0.85 e Å^−^^3^
1409 reflections	Δρ_min_ = −0.76 e Å^−^^3^

### Special details

**Table:**

Geometry. All esds (except the esd in the dihedral angle between two l.s. planes) are estimated using the full covariance matrix. The cell esds are taken into account individually in the estimation of esds in distances, angles and torsion angles; correlations between esds in cell parameters are only used when they are defined by crystal symmetry. An approximate (isotropic) treatment of cell esds is used for estimating esds involving l.s. planes.

### Fractional atomic coordinates and isotropic or equivalent isotropic displacement parameters (Å^2^)

**Table:**

	*x*	*y*	*z*	*U*_iso_*/*U*_eq_	Occ. (<1)
Rb	0.18260 (6)	0.24668 (6)	0.22827 (9)	0.0316 (3)	
Zn1	0.87122 (6)	0.55912 (6)	0.11383 (9)	0.0169 (3)	0.5
Fe1	0.87122 (6)	0.55912 (6)	0.11383 (9)	0.0169 (3)	0.5
Zn2	0.92406 (6)	0.12098 (6)	−0.05652 (9)	0.0166 (3)	0.5
Fe2	0.92406 (6)	0.12098 (6)	−0.05652 (9)	0.0166 (3)	0.5
P1	0.14761 (12)	0.06205 (13)	−0.08572 (19)	0.0166 (4)	
O11	0.1420 (4)	−0.0526 (4)	−0.0852 (6)	0.0295 (12)	
O12	0.2450 (3)	0.1026 (4)	0.0096 (6)	0.0243 (11)	
O13	0.3570 (5)	0.3996 (5)	0.2456 (6)	0.0397 (15)	
O14	0.0638 (4)	0.1055 (5)	−0.0151 (7)	0.0385 (14)	
P2	0.92645 (12)	0.36174 (12)	−0.03358 (18)	0.0144 (4)	
O21	0.8903 (5)	0.2550 (4)	−0.0146 (7)	0.0323 (13)	
O22	0.0389 (4)	0.3613 (4)	−0.0253 (6)	0.0269 (11)	
O23	0.3731 (4)	0.0942 (5)	0.3168 (6)	0.0367 (14)	
O24	0.8990 (4)	0.4217 (4)	0.0972 (6)	0.0252 (11)	

### Atomic displacement parameters (Å^2^)

**Table:**

	*U*^11^	*U*^22^	*U*^33^	*U*^12^	*U*^13^	*U*^23^
Rb	0.0383 (5)	0.0321 (5)	0.0259 (4)	0.0006 (3)	0.0097 (3)	−0.0044 (3)
Zn1	0.0197 (4)	0.0151 (5)	0.0149 (4)	0.0019 (3)	0.0006 (3)	−0.0023 (3)
Fe1	0.0197 (4)	0.0151 (5)	0.0149 (4)	0.0019 (3)	0.0006 (3)	−0.0023 (3)
Zn2	0.0194 (5)	0.0149 (5)	0.0149 (4)	−0.0013 (3)	0.0021 (3)	−0.0026 (3)
Fe2	0.0194 (5)	0.0149 (5)	0.0149 (4)	−0.0013 (3)	0.0021 (3)	−0.0026 (3)
P1	0.0191 (8)	0.0138 (8)	0.0156 (8)	−0.0026 (7)	−0.0004 (6)	0.0035 (6)
O11	0.043 (3)	0.014 (3)	0.033 (3)	−0.003 (2)	0.013 (2)	0.003 (2)
O12	0.019 (2)	0.023 (3)	0.028 (3)	−0.0014 (19)	−0.003 (2)	−0.003 (2)
O13	0.059 (4)	0.043 (3)	0.015 (3)	−0.011 (3)	0.002 (3)	0.011 (2)
O14	0.021 (3)	0.042 (3)	0.052 (4)	0.000 (2)	0.007 (3)	−0.017 (3)
P2	0.0211 (9)	0.0095 (8)	0.0127 (8)	−0.0020 (6)	0.0031 (6)	0.0001 (6)
O21	0.053 (4)	0.016 (3)	0.034 (3)	−0.010 (2)	0.024 (3)	−0.010 (2)
O22	0.023 (3)	0.023 (3)	0.037 (3)	0.000 (2)	0.010 (2)	0.006 (2)
O23	0.037 (3)	0.056 (4)	0.016 (3)	−0.006 (3)	0.004 (2)	−0.011 (2)
O24	0.040 (3)	0.014 (2)	0.023 (3)	0.005 (2)	0.009 (2)	−0.0051 (19)

### Geometric parameters (Å, º)

**Table:**

Rb—O21^i^	2.925 (6)	Zn1—O22^vi^	1.900 (5)
Rb—O12	2.979 (5)	Zn2—O13^vii^	1.860 (6)
Rb—O14	3.098 (6)	Zn2—O14^viii^	1.878 (5)
Rb—O13	3.107 (6)	Zn2—O21	1.897 (5)
Rb—O22	3.109 (5)	Zn2—O11^ix^	1.919 (5)
Rb—O24^i^	3.123 (5)	P1—O13^iii^	1.514 (5)
Rb—O11^ii^	3.181 (5)	P1—O14	1.519 (6)
Rb—O12^iii^	3.215 (6)	P1—O11	1.527 (5)
Rb—O23	3.269 (6)	P1—O12	1.535 (5)
Rb—O21^iv^	3.298 (7)	P2—O22^viii^	1.517 (5)
Zn1—O23^v^	1.877 (5)	P2—O23^vii^	1.520 (5)
Zn1—O24	1.879 (5)	P2—O24	1.523 (5)
Zn1—O12^v^	1.886 (5)	P2—O21	1.522 (5)
			
O21^i^—Rb—O12	142.06 (14)	O12^iii^—Rb—O23	102.79 (14)
O21^i^—Rb—O14	115.16 (17)	O21^i^—Rb—O21^iv^	76.81 (19)
O12—Rb—O14	47.17 (13)	O12—Rb—O21^iv^	98.31 (14)
O21^i^—Rb—O13	108.19 (16)	O14—Rb—O21^iv^	139.07 (14)
O12—Rb—O13	98.37 (15)	O13—Rb—O21^iv^	54.77 (14)
O14—Rb—O13	136.49 (16)	O22—Rb—O21^iv^	148.78 (13)
O21^i^—Rb—O22	110.80 (16)	O24^i^—Rb—O21^iv^	89.55 (14)
O12—Rb—O22	92.90 (15)	O11^ii^—Rb—O21^iv^	80.58 (14)
O14—Rb—O22	66.86 (16)	O12^iii^—Rb—O21^iv^	98.09 (13)
O13—Rb—O22	94.89 (14)	O23—Rb—O21^iv^	44.69 (13)
O21^i^—Rb—O24^i^	47.19 (13)	O23^v^—Zn1—O24	110.7 (3)
O12—Rb—O24^i^	169.12 (13)	O23^v^—Zn1—O12^v^	104.6 (2)
O14—Rb—O24^i^	128.20 (14)	O24—Zn1—O12^v^	115.9 (2)
O13—Rb—O24^i^	79.99 (16)	O23^v^—Zn1—O22^vi^	112.0 (3)
O22—Rb—O24^i^	76.59 (15)	O24—Zn1—O22^vi^	110.8 (2)
O21^i^—Rb—O11^ii^	56.47 (13)	O12^v^—Zn1—O22^vi^	102.6 (2)
O12—Rb—O11^ii^	85.60 (14)	O13^vii^—Zn2—O14^viii^	118.1 (3)
O14—Rb—O11^ii^	76.11 (17)	O13^vii^—Zn2—O21	103.5 (3)
O13—Rb—O11^ii^	135.33 (15)	O14^viii^—Zn2—O21	109.7 (3)
O22—Rb—O11^ii^	129.51 (14)	O13^vii^—Zn2—O11^ix^	110.9 (3)
O24^i^—Rb—O11^ii^	103.19 (13)	O14^viii^—Zn2—O11^ix^	113.5 (3)
O21^i^—Rb—O12^iii^	139.13 (14)	O21—Zn2—O11^ix^	98.8 (2)
O12—Rb—O12^iii^	78.67 (16)	O13^iii^—P1—O14	111.5 (4)
O14—Rb—O12^iii^	95.38 (16)	O13^iii^—P1—O11	110.3 (3)
O13—Rb—O12^iii^	45.51 (13)	O14—P1—O11	109.7 (3)
O22—Rb—O12^iii^	55.68 (13)	O13^iii^—P1—O12	106.8 (3)
O24^i^—Rb—O12^iii^	92.85 (14)	O14—P1—O12	105.7 (3)
O11^ii^—Rb—O12^iii^	163.87 (13)	O11—P1—O12	112.8 (3)
O21^i^—Rb—O23	101.14 (15)	O22^viii^—P2—O23^vii^	110.8 (3)
O12—Rb—O23	56.67 (14)	O22^viii^—P2—O24	110.8 (3)
O14—Rb—O23	94.63 (15)	O23^vii^—P2—O24	109.5 (3)
O13—Rb—O23	80.27 (17)	O22^viii^—P2—O21	109.5 (3)
O22—Rb—O23	147.51 (14)	O23^vii^—P2—O21	110.3 (3)
O24^i^—Rb—O23	132.85 (14)	O24—P2—O21	105.7 (3)
O11^ii^—Rb—O23	64.93 (15)		

Symmetry codes: (i) −*x*+1, *y*, −*z*+1/2; (ii) *x*, −*y*, *z*+1/2; (iii) −*x*+1/2, −*y*+1/2, −*z*; (iv) *x*−1/2, −*y*+1/2, *z*+1/2; (v) *x*+1/2, *y*+1/2, *z*; (vi) −*x*+1, −*y*+1, −*z*; (vii) *x*+1/2, −*y*+1/2, *z*−1/2; (viii) *x*+1, *y*, *z*; (ix) −*x*+1, −*y*, −*z*.
